# Do Not Judge a Book by Its Cover: Critical Need for Longitudinal Neurodevelopmental Assessment of In Utero Zika-Exposed Children

**DOI:** 10.4269/ajtmh.20-0197

**Published:** 2020-04-06

**Authors:** Sarah B. Mulkey, Roberta L. DeBiasi

**Affiliations:** 1 Children’s National Hospital, Washington, District of Columbia;; 2 Department of Pediatrics, The George Washington University School of Medicine and Health Sciences, Washington, District of Columbia;; 3 Department of Neurology, The George Washington University School of Medicine and Health Sciences, Washington, District of Columbia;; 4 Department of Tropical Medicine and Infectious Disease, The George Washington University School of Medicine and Health Sciences, Washington, District of Columbia

Although congenital Zika syndrome has faded from the spotlight for many in the general public, those of us involved in long-term neurodevelopmental follow-up of the children exposed to Zika virus (ZIKV) during the epidemic of 2015–2016 remain concerned for the outcome of children with both recognized and unrecognized ZIKV exposure and infection. Given that as many as 80% of women with Zika infection may be asymptomatic, the magnitude of exposed children is likely far greater than those currently under surveillance. Meanwhile, as the ZIKV story continues to unfold, there is increasing evidence demonstrating that children with in utero ZIKV exposure are at risk for a spectrum of functional neurologic and developmental adverse outcomes.^1–3^ In addition, infants born in areas with endemic transmission may not only undergo prenatal exposure but also be exposed in the early postnatal period, which could add to the risk of adverse neurodevelopmental outcomes. Without careful developmental screening, these children can go unnoticed, yet have developmental effects from congenital infection. We know that children, including those without known congenital infection, often do not get the recommended developmental screening in childhood. In this post-epidemic period, with decreased Zika transmission and less public awareness, follow-up of these children is now more important than ever.

In a study published in this issue of the *American Journal of Tropical Medicine and Hygiene* by Bertolli and others,^4^ the functional outcomes of a large cohort of children from Paraiba in Northeastern Brazil who had congenital ZIKV exposure are reported. The infants were analyzed based on laboratory and anthropometric criteria for congenital ZIKV disease (small head circumference or disproportionately small head circumference for age). Not surprisingly, children with both laboratory and anthropometric criteria were more likely to have abnormal neurologic outcomes and developmental delay more than children meeting only laboratory or anthropometric criteria.^4^ Of great concern is the observation that more than half of children meeting only laboratory criteria displayed abnormal functional outcomes.^4^ This study, thus, provides further impetus to closely follow neurodevelopmental outcomes for all ZIKV-exposed infants through early childhood.

Depending on location and available resources, there are additional challenges to completing neurodevelopmental screening. It is important for developmental assessment tools to be culturally appropriate, translatable to the native language, and able to be administered by providers without formal training in neurodevelopmental assessment.^5^ In addition, normative data specific to the community being studied are often not available but are important to consider, as socioeconomic status and other community factors can greatly influence neurodevelopment. The study by Bertolli and others used the Ages and Stages questionnaire, which has been translated into more than 20 languages and has been used in a similar cohort of Brazilian non–ZIKV-exposed children.^4,6^ Questionnaires like this one are able to detect developmental delays in multiple domains of development, which can then be used to identify resources and therapies to promote improved child development. Multidisciplinary health brigades in underserved areas can also be as an effective method for providing long-term follow-up of Zika-affected infants.^7^ Video- or telemedicine-based methods of mobility testing could build on this success by allowing subspecialty providers who are not able to be physically present to remotely and accurately assess motor and other neurodevelopmental skills.^1^ Using technology to bring specialty providers into remote global communities should be a goal to enhance child neurodevelopmental surveillance for both ZIKV and other emerging infectious and noninfectious threats.

As children with prenatal ZIKV exposure age, our knowledge about the long-term impact of the infection on their outcomes will continue to expand. The use of animal models such as the macaque, with a faster rate of neurodevelopment than the human, can potentially provide us an accelerated insight into future neurologic outcomes for children.^8^ Pooling data and sharing our experience in the evaluation and follow-up of ZIKV child cohorts in multiple regions can also help advance our understanding of the spectrum of neurodevelopmental outcomes.^9^ Future studies need to continue to follow long-term functional and neurodevelopmental outcomes across the full spectrum of children with congenital ZIKV exposure.

A major remaining limitation for clinical evaluation is in the domain of ZIKV laboratory testing. Infant serologic neutralizing antibody positivity before 18 months of age may reflect maternal antibody transfer or, in endemic areas, could reflect postnatal acquisition. Many infants do not have nucleic acid testing performed, and of those that do, many have negative tests, despite confirmed maternal testing during pregnancy. Thus, laboratory confirmation of infection may not be possible in every case, and serologic testing may not differentiate the timing of infection. In an area of endemic ZIKV exposure, a child who presents with developmental delay, whose mother was asymptomatic during pregnancy and did not have ZIKV testing, could have developmental delay owing to unrecognized prenatal ZIKV infection. Unfortunately, there is not currently a reliable laboratory test to identify such a child. With many women having asymptomatic infection, unidentified children with developmental delay due to ZIKV infection is a real concern.

The Zika story continues to be written. Investments in studies like that described in this issue of the *Journal* that follow children longitudinally^4^ are essential to improve our understanding of the full spectrum of effects that Zika presents to the unborn child.

## References

[1] Mulkey SB 2020 Neurodevelopmental abnormalities in children with in utero Zika virus exposure without congenital Zika syndrome. JAMA Pediatr 174: 269–276. 10.1001/jamapediatrics.2019.5204 PMC6990858 31904798

[2] Nielsen-Saines K 2019 Delayed childhood neurodevelopment and neurosensory alterations in the second year of life in a prospective cohort of ZIKV-exposed children. Nat Med 25: 1213–1217. 31285631 10.1038/s41591-019-0496-1 PMC6689256

[3] Pecanha PM 2020 Neurodevelopment of children exposed intra-uterus by Zika virus: a case series. PLoS One 15: e0229434. 32109947 10.1371/journal.pone.0229434 PMC7048286

[4] Bertolli J 2020 Functional outcomes among a cohort of children in northeastern Brazil meeting criteria for follow-up of congenital Zika virus infection. Am J Trop Med Hyg 102: 955–963. 10.4269/ajtmh.19-0961 PMC7204564 32228785

[5] K Connery A 2019 Responding to the Zika epidemic: preparation of a neurodevelopmental testing protocol to evaluate young children in rural Guatemala. Am J Trop Med Hyg 100: 438–444. 30594262 10.4269/ajtmh.18-0713 PMC6367612

[6] Santana CM Filgueiras A Landeira-Fernandez J, 2015 Ages & stages questionnaire-Brazil-2011: adjustments on an early childhood development screening measure. Glob Pediatr Health 2: 2333794X15610038. 10.1177/2333794X15610038 PMC4784636 27335984

[7] Hillman B 2019 2018 U.S. Virgin islands Zika health brigade: providing recommended pediatric health screenings for infants born to mothers with laboratory evidence of Zika virus exposure during pregnancy. Birth Defects Res 111: 360–362. 30856278 10.1002/bdr2.1486

[8] Mavigner M 2018 Postnatal Zika virus infection is associated with persistent abnormalities in brain structure, function, and behavior in infant macaques. Sci Transl Med 10: eaao6975. 29618564 10.1126/scitranslmed.aao6975 PMC6186170

[9] Wilder-Smith A 2019 Understanding the relation between Zika virus infection during pregnancy and adverse fetal, infant and child outcomes: a protocol for a systematic review and individual participant data meta-analysis of longitudinal studies of pregnant women and their infants and children. BMJ Open 9: e026092. 10.1136/bmjopen-2018-026092 PMC6588966 31217315

